# CGREF1 modulates osteosarcoma proliferation by regulating the cell cycle through the Wnt/β-catenin signaling pathway

**DOI:** 10.1186/s10020-024-01038-9

**Published:** 2024-12-20

**Authors:** Zicheng Wei, Kezhou Xia, Wenda Liu, Xinghan Huang, Zhun Wei, Weichun Guo

**Affiliations:** Department of Orthopedics, Renmin Hospital of Wuhan University, 238 Jiefang Road, Wuhan, Hubei Province 430060 China

**Keywords:** Osteosarcoma, CGREF1, Proliferation, Wnt/β-catenin signaling pathway, Cell cycle

## Abstract

**Background:**

Osteosarcoma, the most prevalent primary bone malignancy in children and adolescents, exhibits high heterogeneity. The CGREF1 gene encodes a novel 301 amino acid classical secreted protein that contains the presumed N-terminal signaling peptide and EF hand motif. However, its role in osteosarcoma remains unclear.

**Methods:**

Tumor Immune Estimation Resource (TIMER), The Cancer Genome Atlas (TCGA) and Gene Expression Omnibus (GEO) databases were utilized for bioinformatics analysis. Western blot and immunohistochemistry (IHC) techniques were employed to detect the expression of relevant proteins. siRNA, lentivirus, and plasmid technologies were applied to modulate gene expression. The downstream pathway of CGREF1 was identified through RNA sequencing analysis. Cell counting kit-8 (CCK-8) assay, colony formation assay, flow cytometry, wound healing assay, and Transwell assay were conducted for in vitro functional experiments. In vivo experiments involved subcutaneous tumor formation in nude mice.

**Results:**

Our analysis of public databases and clinical samples revealed that CGREF1 is highly expressed in osteosarcoma and is associated with poor prognosis. Knockdown of CGREF1 impeded cell cycle progression and suppressed the proliferation of osteosarcoma cells. Conversely, upregulation of CGREF1 exhibited an opposing pattern. The RNA-seq data from 143B cells was subjected to analysis, revealing that the differentially expressed genes were predominantly enriched in the Wnt signaling pathway. Further experimental results demonstrated that CGREF1 affects activation of the Wnt pathway by regulating GSK3/β-catenin signaling, thereby affecting proliferation ability of osteosarcoma cells. Finally, experiments using subcutaneous transplanted tumor models in nude mice showed that CGREF1 knockdown inhibited tumor growth in vivo by inhibiting the Wnt/β-catenin signaling pathway.

**Conclusion:**

The expression of CGREF1 was significantly upregulated in osteosarcoma and correlated with unfavorable prognosis. CGREF1 exerted a regulatory effect on the proliferation of osteosarcoma cells both in vitro and in vivo through modulation of the wnt/β-catenin signaling pathway. In the future, targeting CGREF1 could potentially offer a novel therapeutic strategy for treating osteosarcoma.

**Supplementary Information:**

The online version contains supplementary material available at 10.1186/s10020-024-01038-9.

## Introduction

Osteosarcoma, the most common primary bone malignancy, is a type of tumor originating from undifferentiated bone fibrous tissue, usually occurring in the distal femur and proximal tibia(Ritter et al. 2010). The incidence of osteosarcoma in the general population is 2–3/ million/year, accounting for 0.2% of malignant tumor diseases, of which children and adolescents account for about 70%, and the peak of the incidence is consistent with the growth spurt in adolescence(Gill and Gorlick 2021; Mirabello et al. 2009). Due to the low incidence of osteosarcoma, the symptoms are not obvious, and it is not easy to be detected in the early stage, coupled with its high proliferation capacity and aggressiveness, most patients are often relatively severe when diagnosed. Compared to decades ago, the 5-year survival rate for patients with osteosarcoma after standard treatment (including extensive surgical resection, neoadjuvant chemotherapy, and adjuvant chemotherapy) has increased from less than 20% to about 65%(Berner et al. 2015). However, due to the high proliferation and invasive ability of osteosarcoma, its recurrence rate is as high as 35%(Isakoff et al. 2015). In recent decades, with the advancement of medical technology, new treatments for OS (osteosarcoma) (immunotherapy, etc.) have been continuously developed, but the 5-year survival rate of clinical patients has not been significantly improved, which may be related to the high heterogeneity of osteosarcoma(Gill and Gorlick 2021; Smith et al. 2010; Smrke et al. 2021). Therefore, it is of great clinical significance to find out relatively ideal new therapeutic targets for osteosarcoma to improve the treatment strategy for osteosarcoma patients, improve the prognosis and prolong the survival of osteosarcoma patients.

CGREF1 (cell growth regulator with EF-hand domain 1) is a classically secreted protein containing 301 amino acids, including a putative N-terminal signaling peptide and EF-hand motif(Deng et al. 2015). The CGREF1 gene is homologous to the rat Cgr11 gene, which has been shown to edit secreted proteins involved in cell adhesion(Devnath et al. 2009). Initial studies have shown that CGREF1 plays an important role in the regulation of AP-1 transcriptional activity and cell proliferation, and its overexpression can inhibit the proliferation of HEK293T (human embryonic kidney cell) and HCT116 (human colon cancer cell)(Deng et al. 2015). At present, there are few studies on CGREF1 in different tumors, mainly in bioinformatics(Xiang et al. 2023; Chen et al. 2022; Zhihao et al. 2023; Moffatt et al. 2021). The role of CGREF1 in the development of osteosarcoma and its mechanism have not been reported.

In this study, we have identified the oncogenic role of CGREF1 in osteosarcoma and investigated its involvement and potential molecular mechanisms in the malignant biological behaviors of osteosarcoma cells through both in vivo and in vitro experiments. Our findings demonstrate that CGREF1 exerts regulatory control over the activation level of the Wnt pathway by modulating GSK3β/β-catenin signal transduction within this pathway, thereby influencing the malignant proliferative behavior of osteosarcoma cells. Consequently, targeting CGREF1 may hold promise as a novel therapeutic approach for clinical management of osteosarcoma.

## Materials and methods

### Sample collection

From 2020 to 2022, a total of twelve groups comprising human normal tissues and osteosarcoma tissues were procured from Renmin Hospital of Wuhan University. Following surgical procedures, the human samples were promptly preserved in liquid nitrogen for subsequent analysis. The acquisition and utilization of these human samples received ethical approval from the Ethics Committee at Renmin Hospital of Wuhan University.

### Bioinformatics analysis

The Tumor Immune Estimation Resource (TIMER) database was utilized to compare the expression levels of CGREF1 across diverse tumor types. The prognostic potential of CGREF1 in osteosarcoma patients was assessed using human samples obtained from The Cancer Genome Atlas (TCGA) database.

### Cell culture

The human osteosarcoma cell lines Saos-2, U2OS, 143B, and MG63 were purchased from Procell Life Science and Technology (Wuhan, China). The human osteoblast cell line hFOB1.19 was purchased from the Cell Bank of the Typical Culture Preservation Committee of the Chinese Academy of Sciences. The hFOB1.19 cells were maintained in DMEM/F12 medium with 0.3 mg/mL G418 from Servicebio Technology (Wuhan, China), and the other cells were cultured in DMEM (Servicebio Technology, Wuhan, China). All cells were supplemented with 10% FBS (Servicebio Technology, Wuhan, China), penicillin (100 U/mL) and streptomycin (100 mg/mL). The hFOB1.19 cells were cultured in 5% CO_2_ at 33.5 °C, and the other cells were cultured in a humidified atmosphere of 5% CO_2_ at 37 °C. CHIR99021 (MedChemExpress, USA) at a concentration of 5µM was used to activate Wnt pathway for subsequent experiments. The Wnt pathway was pharmacologically inhibited using DIF-3 (MedChemExpress, USA) at a concentration of 30µM for subsequent experimental investigations.

### siRNA and plasmid transfection

Specific small interfering RNA (siRNA) targeting CGREF1 and negative control siRNAs were purchased from OBiO Technology (Shanghai, China). MG63 and 143B cells were seeded in 6-well plates, and the cells were transfected with 20 nM siRNAs using Lipofectamine 2000 reagent according to the manufacturer’s instructions. For overexpression experiments, MG63 and 143B cells were transfected with the pcDNA3.1-EGFP-P2A-CGREF1-3FLAG plasmid using Lipofectamine 2000. The siRNA sequences were as follows:

siCGREF1-1: 5’-ACUCUGAAGUGCAGCAUCAGCTT-3’;

siCGREF1-2: 5’-GAGCUGCUGUCCAUGUUGACATT-3’;

siCGREF1-3: 5’-GGGAAACACUGGAGUCUAAGATT-3’;

siNC: 5’-UUCUCCGAACGUGUCACGUTT − 3’.

### Lentivirus infection

Lentivirus shRNAs targeting CGREF1 (shCGREF1) were purchased from OBiO Technology (Shanghai, China). To acquire the MG63 and 143B cell line with stable CGREF1 knockdown (MG63-shCGREF1 and 143B-shCGREF1), MG63 and 143B cells were infected with shCGREF1 lentivirus (MOI of 143B = 20, MOI of MG63 = 100) using 5 µg/mL polybrene transfection reagent (OBiO Technology, Shanghai, China) and cells were selected with 5 µg /mL puromycin (Servicebio Technology, Wuhan, China).

### Western blot

Total cellular proteins were extracted with radioimmunoprecipitation assay (RIPA, Servicebio Technology, China). Phenylmethanesulfonyl fluoride (PMSF, Servicebio Technology, China) was added to the lysate buffer at a ratio of 1:100. Western blot was performed according to standard procedures described previously(Xia et al. 2023). Antibodies against CGREF1 (1:1000, #YT0893, Immunoway, USA), Cyclin D1(1:10000, #26939-1-AP, Proteintech, China), β-catenin (1:1000, #YT0676, Immunoway, USA), GSK3β (1:1000, #YT2082, Immunoway, USA), phospho-β catenin (Ser33) (1:1000, #80067-1-RR, Proteintech, China), phospho-GSK3β (Ser389) (1:1000, #14850-1-AP, Proteintech, China), β-actin (1:3000, #GB15003, Servicebio Technology, China), Rabbit IgG (1:10000, #BL003A, Biosharp, Canada) were used.

### Cell viability assay

The cell suspension was diluted to 4000 cells/ml and uniformly inoculated into 96-well plates with 100 µl per well. After cell adhesion, 10 µl Cell Counting Kit-8 (CCK-8, Glpbio, USA) was added to each well at 0, 24, 48 and 72 h, respectively. After incubation at 37℃ for 2 h, the absorbance of the solution at 450 nm wavelength was detected with an microplate reader (Thermo Fisher, USA).

### Cell colony formation assay

Cells were uniformly inoculated into 6-well flat plates with a density of 400 cells/well. Each well was cultured with 2 ml DMEM medium containing 10% FBS. It was cultured in a 5% CO_2_ incubator at 37℃ for 10 days, during which the liquid was changed every 2 days. After 10 days of culture, cells were fixed with polymethanol and stained with crystal violet. Count colonies containing > 50 cells under microscope.

### Wound-healing assay

Cell migration was detected by wound healing assay. In simple terms, after the cells are uniformly inoculated on a 6-well plate and cultured to a density of 90 to 100%, a 200 µL sterile pipette tip is applied directly across the cell layer. The cells were washed with PBS 3 times, and after the floating cells were removed, the serum-free medium was added. An inverted microscope (Olympus, Japan) was used to collect images of scratch sites at 0 h and 24 h, and the ImageJ software was used for measurement and analysis.

### Transwell invasion assay

Invasion assays were conducted using Transwell chambers (Corning, USA) and Matrigel (Corning, USA). The bottom of the upper chamber of Transwell was coated with Matrigel (dilution ratio of Matrigel to serum-free medium was 1:6). 5 × 10^4^ cells were inoculated with serum-free medium in the upper chamber of Transwell, and 500µL 20% FBS complete medium was added to the lower chamber. After incubation for 48 h, the Transwell chamber was washed with PBS, fixed with 4% paraformaldehyde for 30 min, and stained with 0.1% crystal violet for 10 min. The invading cells from the upper chamber were observed under an inverted microscope (Olympus, Japan) and counted using the ImageJ software.

### Flow cytometry (FCM) for cell cycle assay

Cell Cycle and Apoptosis Analysis Kit (Servicebio Technology, China) was used to detect cell cycle. 5 × 10^5^ adherent cells were digested and collected, washed and centrifuged with PBS, and 1 mL of 75% ethanol was added to the cell precipitation and fixed at 4℃ for 1 h. After fixation, the cells were washed with PBS, and the working solution (containing RNaseA reagent and PI dye solution) was dyed according to the reagent instructions, and incubated at 37℃ for 30 min without light. Beckman flow cytometry (Beckman Coulter, USA) was used to detect red fluorescence and light scattering at 488 nm excitation wavelength. Data processing using FlowJo v10.

### Immunohistochemistry (IHC) analysis

Following the deparaffinization of the paraffin-embedded tissue sections, sections were incubated with antibody against CGREF1 (1:100, #YT0893, Immunoway), β-catenin (1:100, #YT0676, Immunoway), Ki-67 (1:100, #ab16667, Abcam), Cyclin D1(1:1000, #26939-1-AP, Proteintech, China) at 4 °C overnight. The sections were then cleaned and incubated at 37℃ for 1–2 h with secondary antibodies. DAB Color Development Kit (Servicebio Technology, China) was used for color development. The primary distribution locations (expression patterns) of the relevant proteins: CGREF1- cytoplasm, β-catenin-cytoplasm/cytomembrane/nucleus, Ki-67-nucleus, Cyclin D1-nucleus. Images were taken using an inverted microscope (Olympus, Japan) (a 10x eyepiece with a 20x objective). Data processing using the ImageJ software.

### Xenograft tumor model

All studies were approved by the Ethics Committee of the Renmin Hospital of Wuhan University. In this experiment, 4-week-old male BALB/c athymic nude mice were purchased from Shulaibao Biotech (Wuhan, China) and raised in SPF environment. Nude mice were randomly divided into two groups with 6 mice in each group, and 1 × 10^6^ 143B-shCGREF1 or 143B-shNC cells were injected subcutaneously (200µL PBS mixed). They were kept in an SPF environment with adequate food and water for another 4 weeks. Tumor volume was measured every 7 days. The tumor volume was calculated with the formula: (length × width^2^)/2. Changes in diet, mental, activity status, and body weight were observed every two days during rearing, and pain and distress were assessed. After 4 weeks, the mice were killed by euthanasia (by inhalation of excess carbon dioxide) and the tumor volume and weight were measured. Finally, the tumors were preserved in liquid nitrogen or paraformaldehyde for subsequent experiments.

### Statistical analysis

All data are expressed as mean ± standard deviation (SD). Statistical analysis was performed using GraphPad Prism 9.0. Each experiment was repeated three times. Significant differences between the two groups were assessed using two-tailed Student’s t-test. At *P* < 0.05, the difference was considered statistically significant.

## Results

### CGREF1 is highly expressed in osteosarcoma and is associated with poor prognosis

**Fig. 1:**
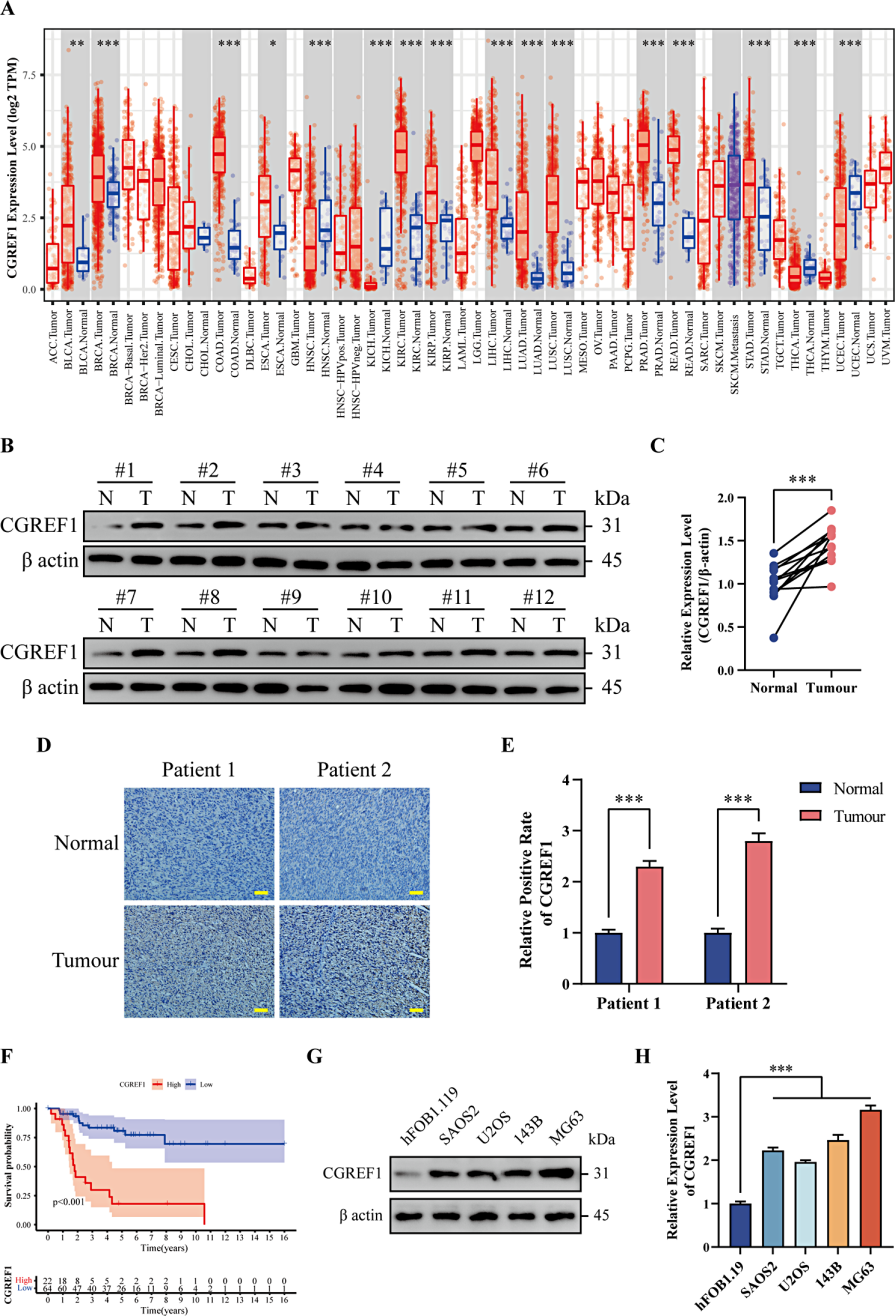
Expression level of CGREF1 in osteosarcoma was correlated with prognostic value for osteosarcoma patients. (**A**) The expression level of CGREF1 in various cancers was analyzed using data from the TIMER database. (**B**-**C**) Western blot assay was performed to detect the expression of CGREF1 in osteosarcoma tissues and adjacent normal tissues, and the results were summarized graphically based on three experiments. (**D**-**E**) Immunohistochemistry staining was conducted to examine the presence of CGREF1 in normal and osteosarcoma tissues, and a histogram format was used to summarize the results obtained from three experiments. (**F**) Kaplan-Meier survival analysis revealed that high expression levels of CGREF1 were associated with poor prognosis among osteosarcoma patients compared to those with low CGREF1 expression. (**G**-**H**) Western blot analysis was employed to determine the protein expression levels of CGREF1 in hFOB1.19, Saos-2, U2OS, 143B, and MG63 cell lines. Results from three experiments are summarized in a histogram format. Scale bar: 200 μm. (*P < 0.05, **P < 0.01, ***P < 0.001)

We initially investigated the levels of CGREF1 in various tumor types and observed a significant upregulation of CGREF1 expression in diverse tumors (including renal clear cell carcinoma, colorectal adenocarcinoma, rectal adenocarcinoma, etc.) compared to their respective non-tumor counterparts (Fig. 1A). We subsequently conducted western blot analysis on 12 osteosarcoma samples and observed a significant upregulation of CGREF1 protein levels in osteosarcoma tissues compared to corresponding non-tumor tissues (Fig. 1B, C). The confirmation of this was further supported by the results from clinical patient IHC analysis (Fig. 1D, E). The survival of osteosarcoma patients in the TCGA database was analyzed using the Kaplan-Meier method. The results revealed a significantly higher survival rate in the group with low CGREF1 expression compared to the group with high CGREF1 expression (Fig. 1F). Subsequently, different osteosarcoma cell lines were screened for their CGREF1 expression levels, and two cell lines, namely 143B and MG63, exhibiting relatively high CGREF1 expression were selected for subsequent experiments (Fig. 1G, H).

### Silence CGREF1 blockade of osteosarcoma cells of the cell cycle, inhibit cell proliferation

**Fig. 2:**
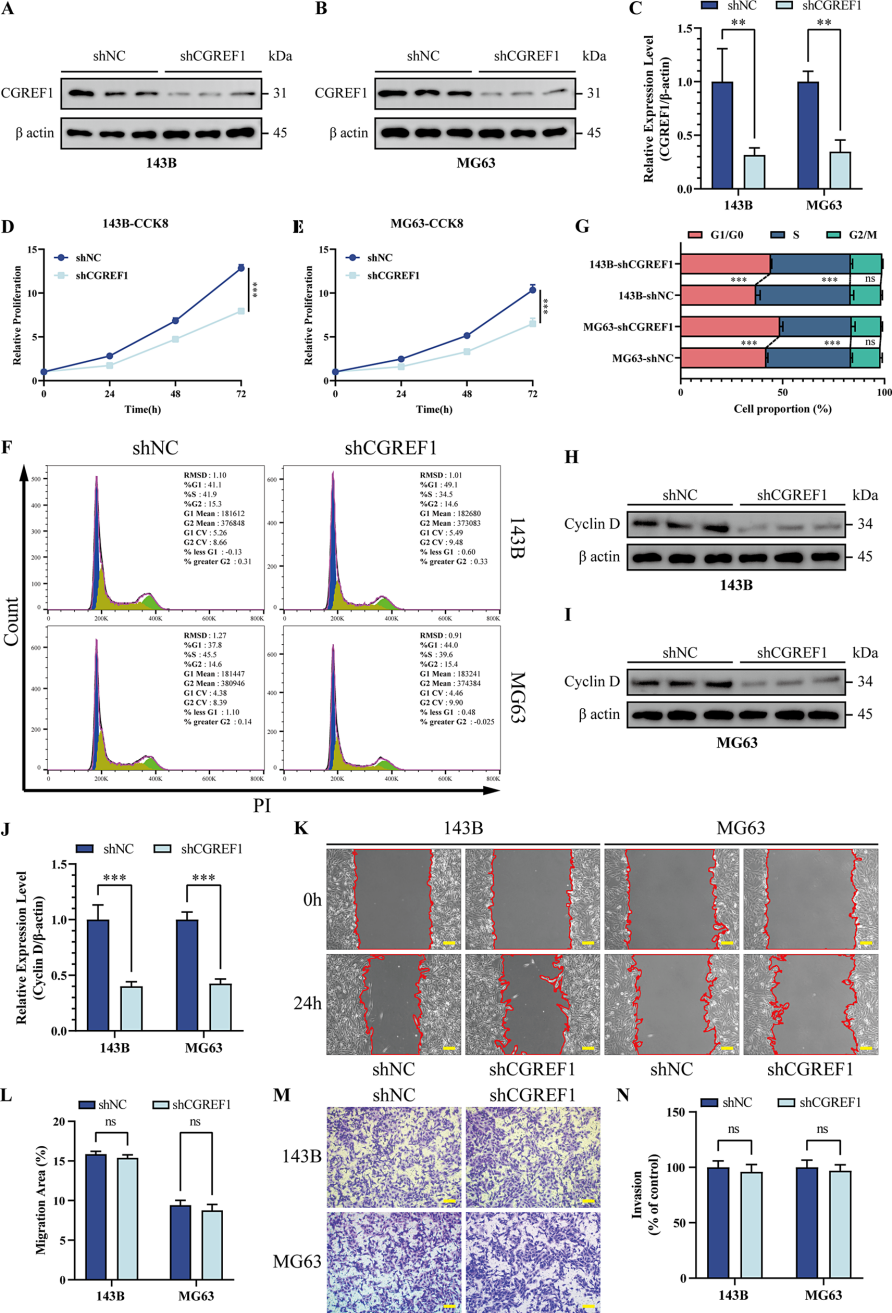
Silence CGREF1 blockade of osteosarcoma cells of the cell cycle, inhibit cell proliferation. (**A**-**C**) Western blot analysis confirmed a significant decrease in the expression of the CGREF1 gene in both shCGREF-143B and shCGREF1-MG63 cell lines. Results from three experiments are summarized in a histogram format. (**D**-**E**) The CCK-8 assay was employed to assess the impact of CGREF1 knockdown on cell proliferation stability. (**F**-**G**) Flow cytometry was utilized to examine the cell cycle distribution in 143B-shCGREF1 and MG63-shCGREF1 cells. The findings of three experiments were succinctly presented in the form of a histogram. (**H**-**J**) Western blot analysis confirmed that the knockdown of CGREF1 resulted in a significant decrease in Cyclin D expression. Results from three experiments are summarized in a histogram format. (**K**-**L**) Wound healing assay was performed to evaluate the migratory ability of stable CGREF1-knockdown cells compared to control cells. Results from three experiments are summarized in a histogram format. (**M**-**N**) Transwell invasion experiment was conducted to investigate the effect of low stability due to CGREF1 knockdown on cellular invasiveness relative to control group. Results from three experiments are summarized in a histogram format. Scale bar: 200 μm. (*P < 0.05, **P < 0.01, ***P < 0.001)

Given the high expression of CGREF1 in osteosarcoma cell lines 143B and MG63, we designed three siRNAs targeting CGREF1 for transfection into these cell lines, followed by assessment of knockdown efficiency using western blot analysis. Our results demonstrate that siCGREF1-1 exhibits the most pronounced knockdown efficacy (Supplementary Fig. 1A, B, D, E). CCK8 experiments following siRNA transfection demonstrated a reduction in osteosarcoma cell proliferation at lower levels of CGREF1 knockdown, directly proportional to the extent of knockdown (Supplementary Fig. 1 C, F). Subsequently, stable cell lines of 143B and MG63 with low CGREF1 expression were generated through lentivirus infection using the siCGREF1-1 sequence, and the efficiency of stable knockdown was confirmed to be low. The Western blot results demonstrated the successful establishment of stable CGREF1 knockdown in 143B and MG63 cell lines (Fig. 2A-C). The CCK8 results from the CGREF1 cell line were consistent with those obtained after siRNA transfection, suggesting weakened proliferation ability of osteosarcoma cells upon CGREF1 knockdown (Fig. 2D, E). Flow cytometry analysis showed that partial blockade of G1/G0 phase to S phase transition occurred upon CGREF1 knockdown in both 143B and MG63 cells resulting in impaired mitosis but had no significant effect on G2/M phase (Fig. 2F, G). The subsequent Western blot analysis of key proteins involved in the transition from G1/G0 to S phase of the cell cycle revealed that knockdown of CGREF1 resulted in a decrease in Cyclin D expression levels (Fig. 2H-J). Cell scratch migration and Transwell invasion experiments demonstrated no significant impact on migration and invasion abilities upon CGREF1 knockdown in both 143B and MG63 osteosarcoma cells (Fig. 2K-N). The overall findings demonstrated that the knockdown of CGREF1 resulted in a reduction in Cyclin D expression levels and perturbed cell cycle progression, leading to decreased cellular proliferation. However, no significant impact on migration and invasion was observed.

### Overexpression of CGREF1 facilitates cell cycle progression and enhances cellular proliferation in osteosarcoma

**Fig. 3:**
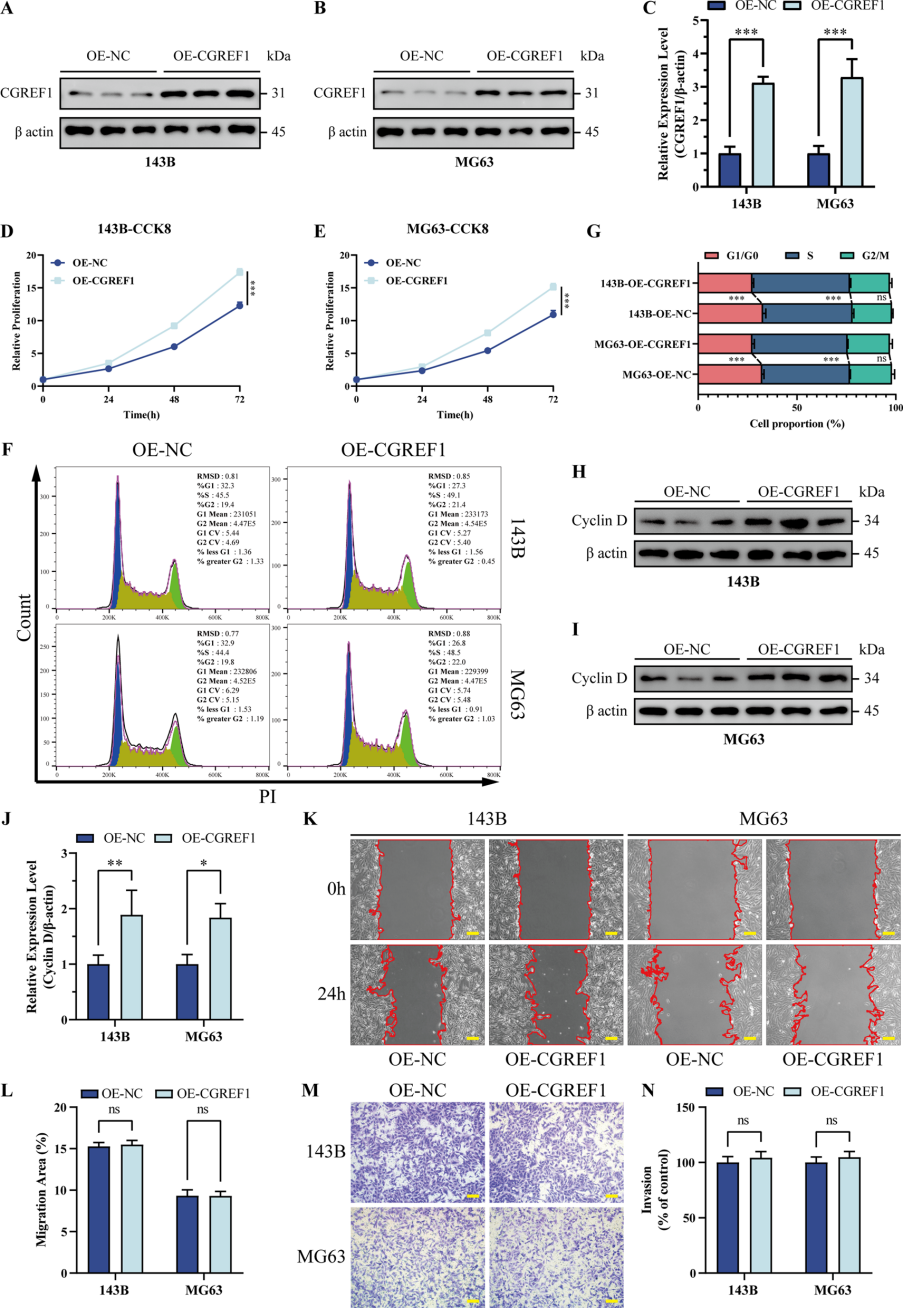
Overexpression of CGREF1 facilitates cell cycle progression and enhances cellular proliferation in osteosarcoma. (**A**-**C**) Western blot analysis validated the upregulation of CGREF1 in 143B and MG63 cell lines. Results from three experiments are summarized in a histogram format. (**D**-**E**) CCK 8 method tested overexpress CGREF1 osteosarcoma cell proliferation ability. (**F**-**G**) Flow cytometry was utilized to examine the cell cycle distribution in 143B-OE-CGREF1 and MG63-OE-CGREF1 cells. The findings of three experiments are succinctly presented in the form of a histogram. (**H**-**J**) Western blot analysis confirmed that the overexpression of CGREF1 resulted in an upregulation of Cyclin D expression. Results from three experiments are summarized in a histogram format. (**K**-**L**) Wound healing assay was performed to evaluate the migratory ability of stable CGREF1-overexpression cells compared to control cells. Results from three experiments are summarized in a histogram format. (**M**-**N**) The Transwell invasion assay was employed to assess the impact of CGREF1 overexpression on cellular invasiveness. Results from three experiments are summarized in a histogram format. Scale bar: 200 μm. (*P < 0.05, **P < 0.01, ***P < 0.001)

Plasmids were employed for transfecting 143B and MG63 cell lines to induce CGREF1 overexpression, which was subsequently confirmed by western blot analysis. The results demonstrated a significant increase in the expression level of CGREF1 in transfected cells (Fig. 3A-C). Moreover, CCK8 assay revealed an augmented proliferation rate of osteosarcoma cells following CGREF1 overexpression (Fig. 3D, E). Flow cytometry analysis further indicated that the upregulation of CGREF1 promoted the transition from G1/G0 phase to S phase in both 143B and MG63 cells (Fig. 3F, G). Notably, Western blot examination exhibited an elevation in Cyclin D expression levels upon CGREF1 overexpression (Fig. 3H-J). However, no substantial impact on migration and invasion ability of osteosarcoma cells was observed after the overexpression of CGREF1 as determined by cell scratch migration and Transwell invasion experiments (Fig. 3K-N). Overall, these findings suggest that elevated levels of CGREF1 enhance Cyclin D expression, accelerate cell cycle progression, and augment proliferative capacity in osteosarcoma cells.

### CGREF1 gene is implicated in the regulation of the wnt signaling pathway

**Fig. 4:**
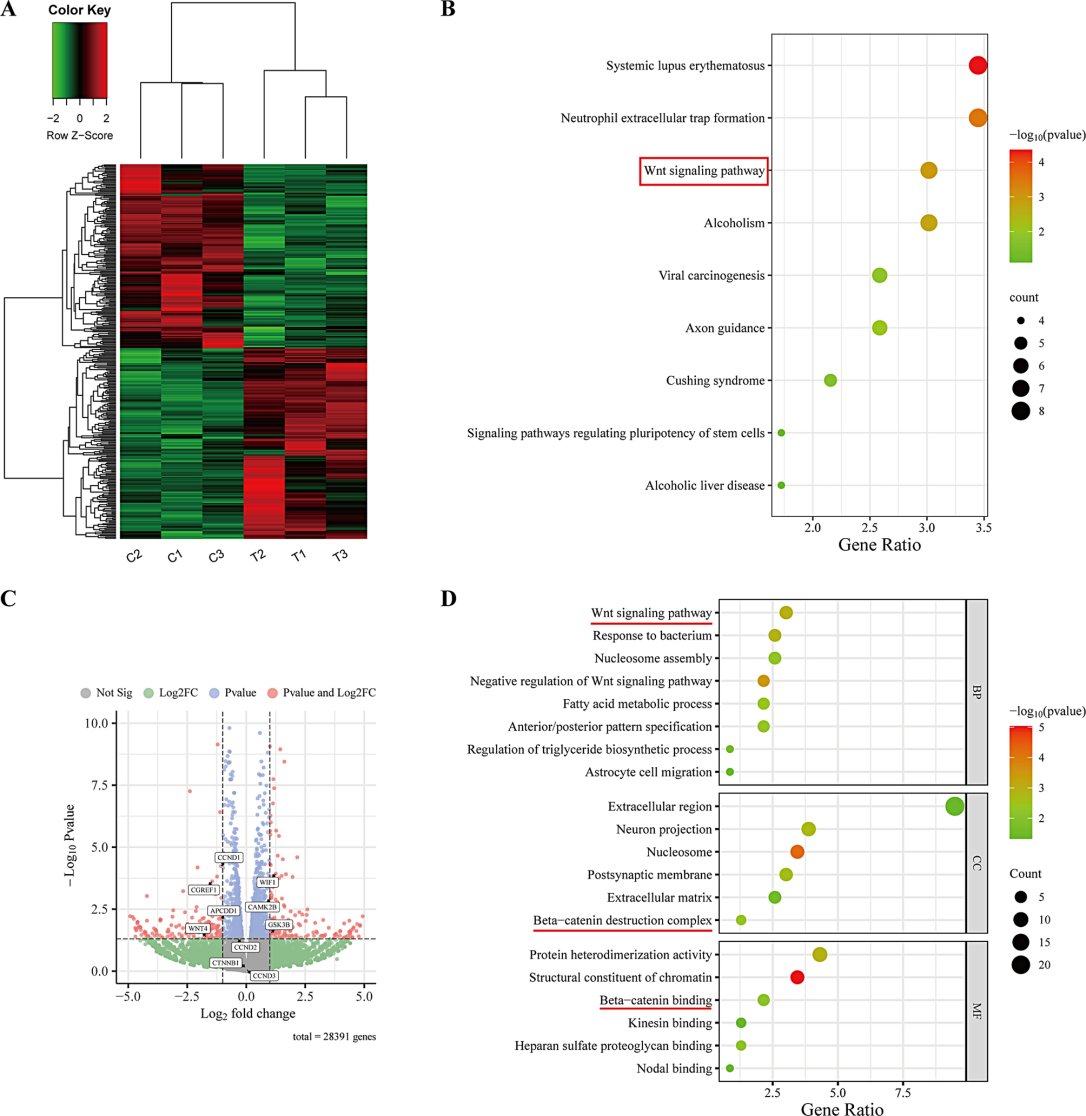
CGREF1 gene is implicated in the regulation of the Wnt signaling pathway. (**A**) Heatmap analysis was used to explore the expression pattern of CGREF1 related differentially expressed genes in RNA-seq data. (**B**) The KEGG enrichment analysis revealed a significant enrichment of CGREF1-associated genes in the Wnt signaling pathway. (**C**) The volcano plot depicted the differential gene expression and the expression relationship between cell cycle-related genes, wnt pathway-related genes and CGREF1 gene. (**D**) The GO enrichment analysis unveiled the involvement of CGREF1 in various cellular components, biological processes, and molecular functions

To explore the underlying molecular mechanism by which CGREF1 exerts its inhibitory effect on osteosarcoma cell proliferation, we performed RNA-Seq analysis following CGREF1 knockdown in 143B cells. A total of 451 differentially expressed genes (DEGs) were identified, with 230 genes up-regulated and 221 genes down-regulated upon CGREF1 knockdown (Fig. 4A, C). KEGG and GO enrichment analyses of the DEGs revealed a significant association between CGREF1 and the wnt signaling pathway, suggesting its potential involvement in β-catenin-mediated molecular functions and biological processes (Fig. 4B, D). KEGG enrichment analysis showed that CGREF1 might affect the neutrophil extracellular trap formation (Fig. 4B). GO analysis showed that in the cellular component part, DEGs may affect the extracellular region (Fig. 4D). Collectively, our findings strongly support a close correlation between CGREF1 function and the wnt signaling pathway.

### CGREF1 affects the malignant proliferation of osteosarcoma cells through modulation of the GSK3β/ β catenin signaling cascade within the Wnt pathway

**Fig. 5:**
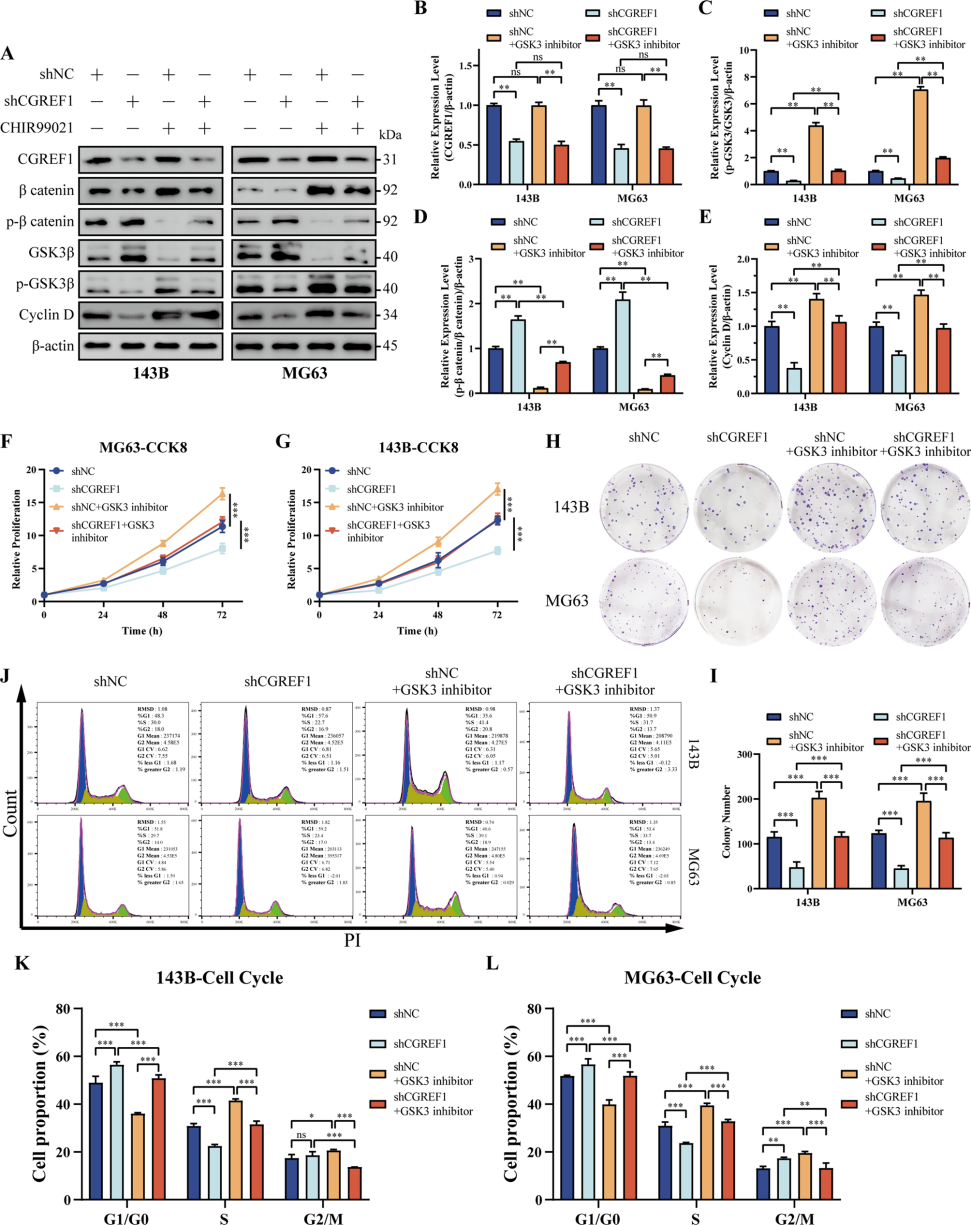
CGREF1 modulates osteosarcoma cell proliferation via the GSK3/β-catenin signaling pathway, and activation of the Wnt signaling pathway can counteract the impact of CGREF1 knockdown on osteosarcoma cells. (**A**-**E**) Western blot analysis was employed to detect the expression levels of CGREF1, Cyclin D, p-GSK3β/GSK3β and p-β catenin/β catenin following CGREF1 knockdown and/or activation of the Wnt pathway. The results from three independent experiments were summarized in a histogram format. (**F**-**G**) CCK8 assays demonstrated that the activation of Wnt pathways attenuated the inhibitory effect of CGREF1 on cell proliferation. (**H**-**I**) Colony formation experiments revealed that the activation of Wnt pathways weakened the suppressive effect of CGREF1 on cell colony formation. The results from three independent experiments were presented concisely in a histogram format. (**J**-**L**) Flow cytometry analysis showed that the activation of the Wnt pathway alleviated the cell cycle arrest induced by CGREF1 knockdown. The findings from three independent experiments were succinctly depicted using histograms. (GSK3 inhibitor: 5µM CHIR99021) (*P < 0.05, **P < 0.01, ***P < 0.001)

In order to further verify the effect of CGREF1 on wnt signaling pathway, we activated wnt signaling pathway related proteins in 143B and MG63 cells with stable CGREF1 knockdown, and conducted gene restoration experiments. Western blot results showed that: After CGREF1 knockdown, p-β catenin/β catenin levels increased, p-GSK3β/GSK3β levels decreased, Cyclin D level decreased, β catenin phosphorylation increased, and GSK3β phosphorylation decreased. After the use of wnt signaling pathway activator (5µM CHIR99021), the expression level of CGREF1 was not affected. However, it could partially restore the levels of wnt signaling pathway-related proteins and the cell cycle protein Cyclin D (Fig. 5A-E). The results of CCK8 and plate cloning showed that CGREF1 knockdown decreased the proliferation ability of osteosarcoma cells. Compared with the control group, the proliferation capacity of osteosarcoma cells was enhanced after the use of wnt signaling pathway activator. Compared with the CGREF1 knockdown group, the reduced cell proliferation induced by CGREF1 knockdown was reversed with the use of wnt signaling pathway activator (Fig. 5F-I). Flow cytometry showed that compared with the control group, the use of wnt signaling pathway activator promoted the transformation of osteosarcoma cells from G1/G0 phase to S phase. Compared with the CGREF1 knockdown group, activation of the wnt signaling pathway could restore the cell cycle blocking effect induced by CGREF1 knockdown (Fig. 5J-L).

**Fig. 6:**
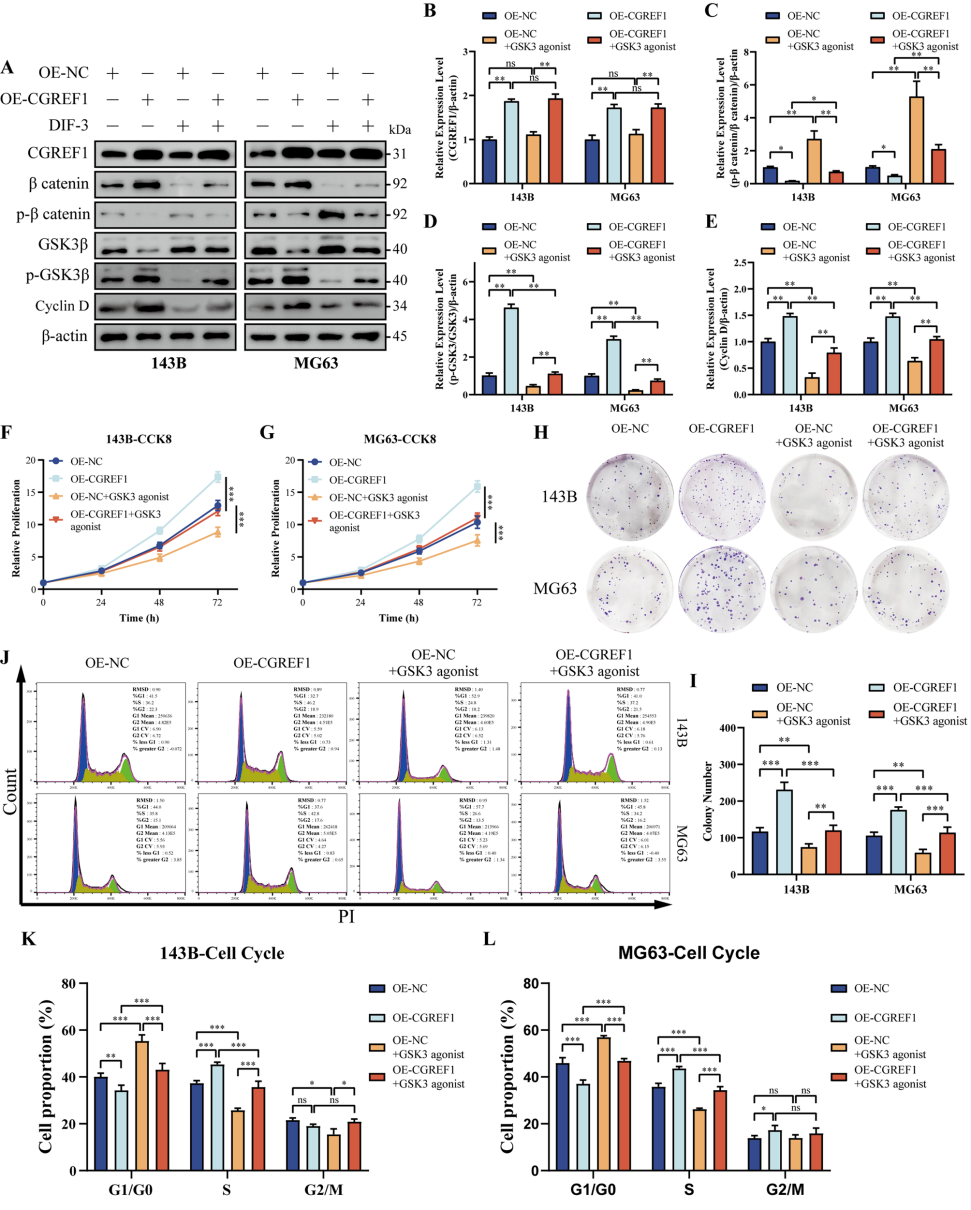
Inhibition of wnt pathway could reverse the effect of CGREF1 overexpression on osteosarcoma cells. (**A**-**E**) Western blot analysis was employed to detect the expression levels of CGREF1, Cyclin D, p-GSK3β/GSK3β and p-β catenin/β catenin following CGREF1 overexpression and/or inhibition of the Wnt pathway. The results from three independent experiments were summarized in a histogram format. (**F**-**G**) The CCK-8 assay demonstrated that inhibition of the Wnt pathway attenuated the promotive effect of CGREF1 overexpression on cellular proliferation. (**H**-**I**) Colony formation assay showed that wnt pathway inhibitor attenuated the promoting effect of CGREF1 overexpression on cell colony formation. The results from three independent experiments were presented concisely in a histogram format. (**J**-**L**) Flow cytometry showed that wnt pathway inhibitor attenuated the cell cycle promotion effect of CGREF1 overexpression. The findings from three independent experiments were succinctly depicted using histograms. (GSK3 agonist: 30µM DIF-3) (*P < 0.05, **P < 0.01, ***P < 0.001)

At the same time, we used wnt signaling pathway inhibitors in osteosarcoma cell lines overexpressing CGREF1 to perform overexpressed gene restoration experiments. Western blot results showed that: Overexpression of CGREF1 decreased the level of p-β catenin/β catenin, reduced the phosphoric acid degradation of β catenin, increased the level of p-GSK3β/GSK3β, increased the phosphorylation level of GSK3β, and increased the level of Cyclin D. The use of wnt signaling pathway inhibitors (30µM DIF-3) did not affect the expression level of CGREF1. However, it could partially restore the levels of wnt signaling pathway-related proteins and the cell cycle protein Cyclin D (Fig. 6A-E). The results of CCK8 and plate cloning showed that overexpression of CGREF1 enhanced the proliferation of osteosarcoma cells. Compared with the control group, the proliferation ability of osteosarcoma cells was weakened after the treatment of wnt signaling pathway inhibitors. Compared with the CGREF1 overexpression group, the enhanced cell proliferation induced by CGREF1 overexpression could be restored after the use of wnt signaling pathway inhibitors (Fig. 6F-I). Flow cytometry showed that wnt signaling pathway inhibitors partially blocked the transition from G1/G0 to S phase of osteosarcoma cells compared with the blank control group. wnt signaling pathway inhibitors after CGREF1 overexpression can reduce the cell cycle promoting effect of CGREF1 overexpression (Fig. 6J-L).

Collectively, these findings suggest that CGREF1 exerts an influence on Cyclin D expression through the regulation of GSK3β/β catenin signaling in the wnt pathway, thereby modulating the malignant proliferation of osteosarcoma cells.

### CGREF1 promotes tumor growth in vivo

**Fig. 7:**
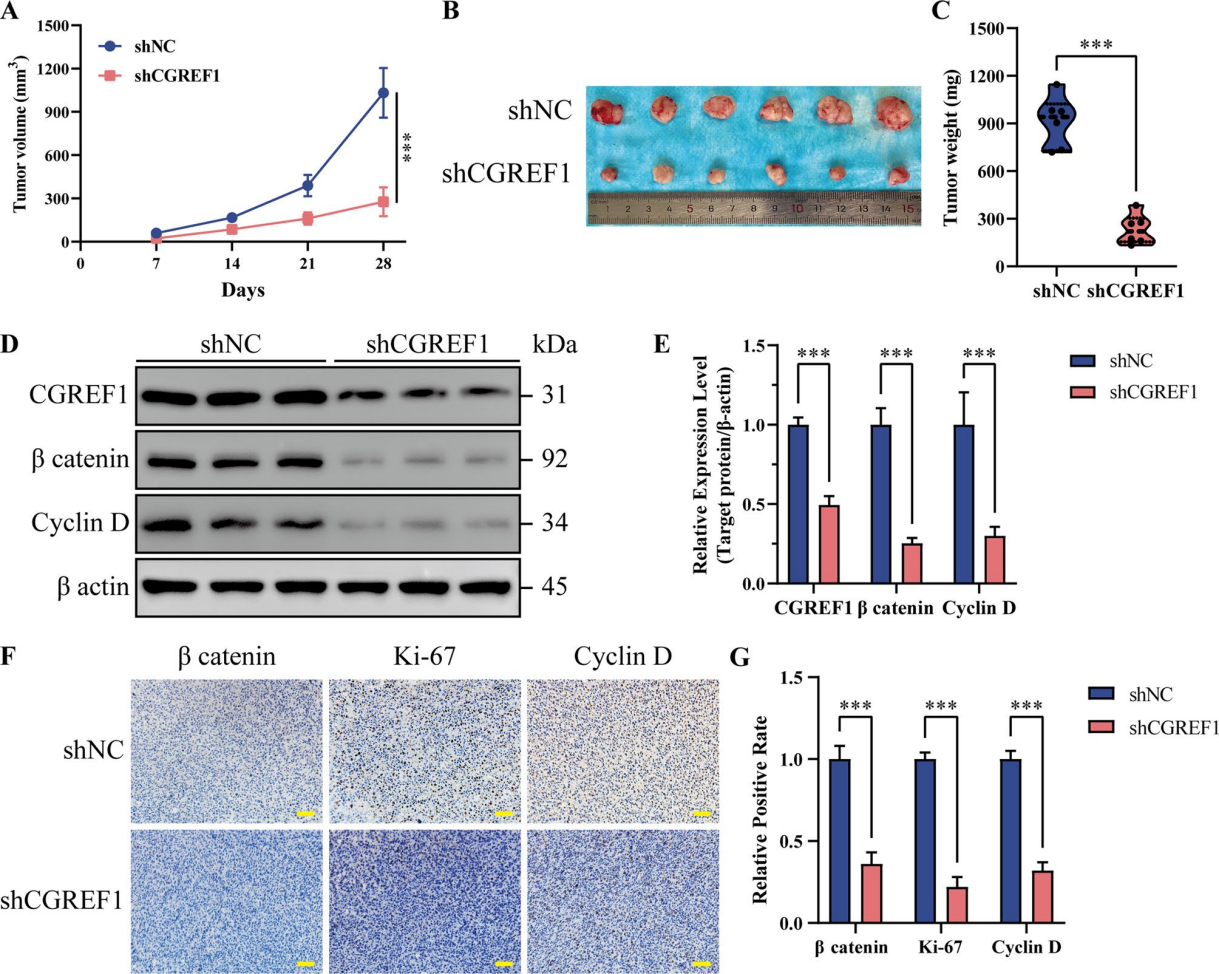
CGREF1 knockdown inhibited the proliferation ability of osteosarcoma cells in vivo. (**A**) The tumor growth curve of nude mice subcutaneously injected with 143B-shCGREF1 and control cells. (**B**) The tumors of the nude mice in the two groups were isolated and photographed. (**C**) The tumors in the nude mice were weighed and compared. (**D**-**E**) Western blot was used to analyze the expression of CGREF1, β-catenin and Cyclin D in tumor tissues of the two groups. The results from three independent experiments were presented concisely in a histogram format. (**F**-**G**) IHC analysis of β catenin, Ki-67 and Cyclin D in xenograft tumors. The results from three independent experiments were presented concisely in a histogram format. Scale bar: 200 μm. (*P < 0.05, **P < 0.01, ***P < 0.001)

To evaluate the biological function of CGREF1 in the development and development of osteosarcoma in vivo, 143B cells (shCGREF1) or control cells (shNC) with stable and low expression of CGREF1 were implanted subcutaneously into male BALB/c nude mice, and tumor detection was performed every 7 days. The results showed that knocking down CGREF1 could significantly inhibit tumor growth. The nude mice were killed 28 days after injection, the tumors were isolated, photographed and weighed. The results showed that the volume and weight of tumors in the CGREF1 knockout group were significantly lower than those in the control group (Fig. 7A-C). Furthermore, western blot analysis revealed a significant reduction in the activation level of the wnt signaling pathway in the CGREF1 knockdown group compared to the control group. Additionally, there was a notable decrease in Cyclin D expression levels observed in the CGREF1 knockdown group when compared to the control group (Fig. 7D, E). IHC results demonstrated that the levels of Ki-67 and Cyclin D in tumor tissues were significantly reduced in the CGREF1 knockdown group compared to the control group, indicating a decrease in cell proliferation. Additionally, there was a notable attenuation of Wnt pathway activation (Fig. 7F, G, Supplementary Fig. 2). In short, CGREF1 enhances tumor formation and growth of osteosarcoma in vivo.

## Discussion

CGREF1 is a classical pathway secreted protein, and initial studies have shown that it plays an important role in the regulation of AP-1 transcriptional activity and cell proliferation(Deng et al. 2015). With the rise of bioinformatics technology, researchers began to focus on the impact of CGREF1 on tumor disease. In the study of prostate cancer anti-tumor genes, CGREF1 and other 21 genes were listed as prostate cancer anti-tumor genes in related studies(Díaz et al. 2022). In a predictive model for overall survival of early-onset colorectal cancer, low CGREF1 expression was associated with poor prognosis(Xiang et al. 2023). In contrast, in cervical cancer studies, nicotinamide adenine dinucleotide (NAD+) metabolic models have shown that high expression of CGREF1 is associated with poor prognosis(Chen et al. 2022). In a study of neuroblastoma patients undergoing chemotherapy, CGREF1 has been identified as a novel somatic mutation during chemotherapy that is involved in mediating nerve cell growth and may be associated with chemotherapy resistance and poor prognosis in neuroblastoma patients(Duan et al. 2018). In studies of cancer associated fibroblasts (CAFs) -associated infiltration models, vasculogenic mimicry (VM) -associated models, oxidative stress-related models, and cisplatin resistance models of osteosarcoma, CGREF1 is considered to be an oncogene, and high expression of CGREF1 is associated with poor prognosis(Zhihao et al. 2023; Yan et al. 2023; Ding et al. 2023; Xie et al. 2023).

Previous studies were mostly based on bioinformatics analysis, but there were few specific studies on the biological function of CGREF1, and CGREF1 showed different prognostic characteristics in different tumors. In this study, analysis of TCGA patient data revealed a significant correlation between high expression levels of CGREF1 and poor prognosis in osteosarcoma patients. Additionally, western blot analysis confirmed the elevated expression of CGREF1 in osteosarcoma samples. Subsequently, we manipulated the gene expression of CGREF1 in osteosarcoma cell lines and conducted comprehensive in vitro functional experiments. Our findings demonstrated that CGREF1 did not directly influence the migration and invasion capabilities of osteosarcoma cells. However, knockdown of CGREF1 resulted in decreased Cyclin D expression, leading to inhibition of cell cycle progression and subsequent suppression of proliferation in osteosarcoma cells. Conversely, overexpression of CGREF1 exerted an opposite effect.

To explore the underlying mechanism of CGREF1 in OS, we performed RNA-seq analysis and found that the differentially expressed genes after CGREF1 knockdown were particularly enriched in wnt signaling pathway and related to the biological process of β-catenin. Previous studies have shown that the Wnt signaling pathway is a key pathway controlling OS development(Ji et al. 2020). The translocation of β-catenin into the nucleus triggers the activation of multiple transcription factors, thereby orchestrating downstream signaling cascades and facilitating the expression of a diverse array of proteins, including Cyclin D(Liu et al. 2022; Zhang and Wang 2020; Yu et al. 2021). Meanwhile, GSK3β can phosphorylate β-catenin, promoting its rapid degradation and preventing the transcription of β-catenin target genes in the cell nucleus(Zeng et al. 2005; Aberle et al. 1997). Although mutations in the Wnt signaling pathway target genes and Wnt signaling pathway-related genes are associated with OS, the precise mechanism by which the Wnt signaling pathway contributes to OS remains a subject of controversy. Some studies have suggested that activation of the Wnt signaling pathway promotes OS growth(Kansara et al. 2009; Ji et al. 2015; Wen et al. 2021; Liu et al. 2018; Matsuoka et al. 2020), while others have come to the opposite conclusion(Cai et al. 2010). In the present study, we demonstrated that knockdown of CGREF1 resulted in inhibition of GSK3β phosphorylation, reduction in β-catenin protein levels, and suppression of downstream Cyclin D activation by β-catenin, consequently leading to impaired proliferation ability of osteosarcoma cells. Conversely, overexpression of CGREF1 exhibited an opposite effect. Moreover, the wnt reversion experiment further substantiated that CGREF1 modulated osteosarcoma cell proliferation through regulation of GSK3β/β-catenin signaling within the wnt pathway. It has been reported that β-catenin is a transcription factor associated with EMT or metastasis, and plays an important role in various tumors(Lv et al. 2016; Gonzalez and Medici 2014; Zhao et al. 2022; Xue et al. 2024). The activation of the wnt/β-catenin signaling pathway contributes to the metastasis of osteosarcoma cells(Wen et al. 2021); however, our study did not demonstrate any impact on the metastatic ability of these cells. This discrepancy may be attributed to the underlying mechanism of CGREF1, which necessitates further exploration and investigation. In the KEGG and GO enrichment analysis of the DEGs, the ratio of genes involved in neutrophil extracellular trap formation was found to be higher compared to those associated with the Wnt signaling pathway. Additionally, a higher ratio of genes related to the extracellular region was observed. This suggests that CGREF1 may have an impact not only on tumor cells themselves but also on other cells (such as neutrophils) within the tumor microenvironment through mechanisms like vesicle secretion and other pathways, which necessitates further exploration and investigation(Zhang et al. 2018, 2024).

## Conclusions

**Fig. 8:**
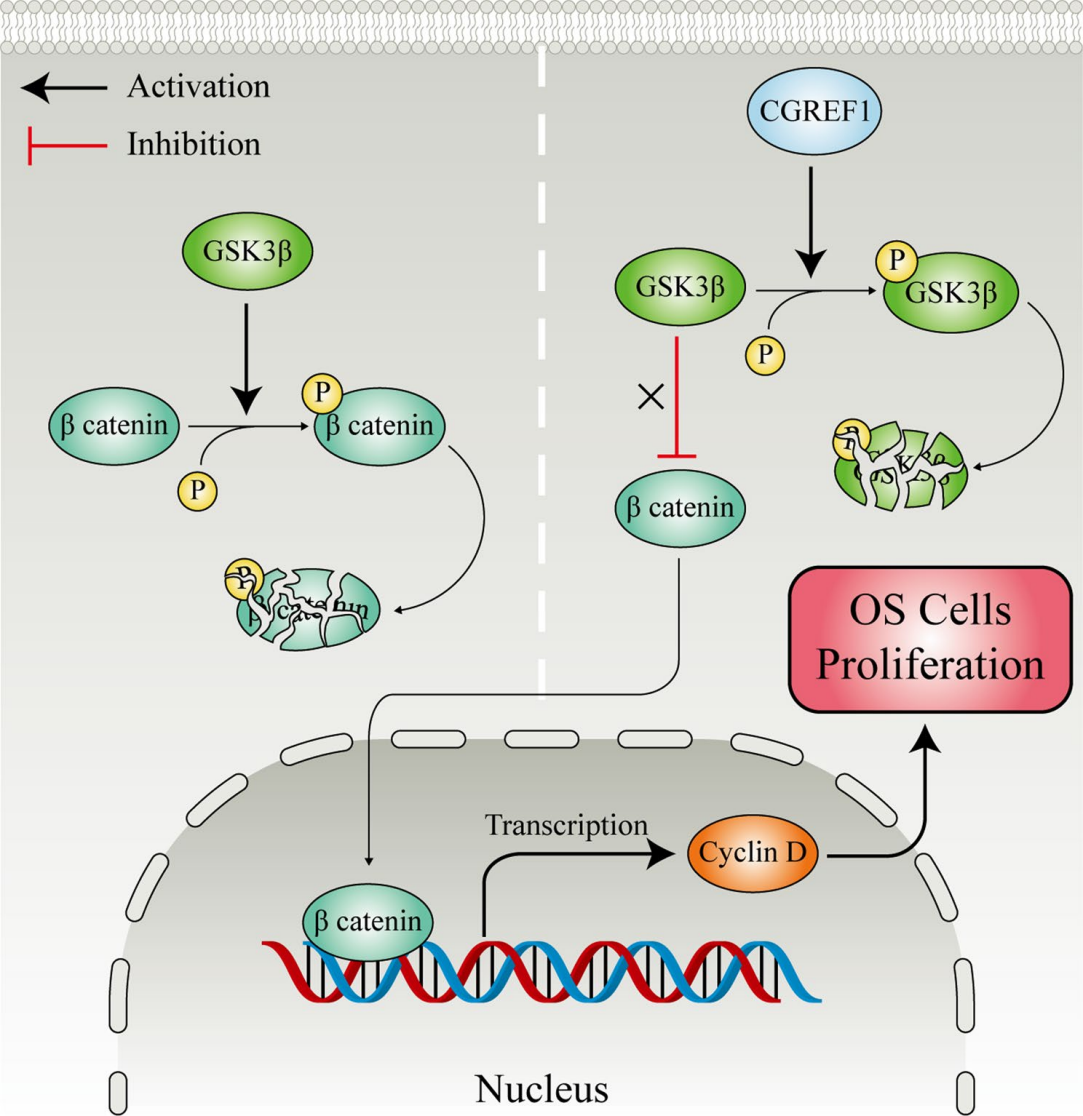
The regulatory mechanisms of CGREF1 on osteosarcoma proliferation

Our study demonstrated that CGREF1 functions as an oncogene in OS. In patients with osteosarcoma, elevated expression of CGREF1 is associated with a higher degree of malignancy and poor prognosis. Mechanistically, CGREF1 enhances Cyclin D expression by modulating GSK3β/β-catenin signaling within the Wnt pathway, thereby promoting the transition of osteosarcoma cells from G1/G0 to S phase and facilitating their proliferation (Fig. 8). Consequently, simultaneous targeting of both CGREF1 and the Wnt signaling pathways may offer novel therapeutic strategies for treating individuals with osteosarcoma.

## Electronic Supplementary Material

Below is the link to the electronic supplementary material.

Supplementary Material 1

## Data Availability

No datasets were generated or analysed during the current study.
