# Supplementary material for: CGREF1 modulates osteosarcoma proliferation by regulating the cell cycle through the Wnt/β-catenin signaling pathway
Source: Mol Med. 2024 Dec 20;30:260. doi: 10.1186/s10020-024-01038-9 (PMC11661040; doi:10.1186/s10020-024-01038-9)
Supplement: Supplementary file 1 — Supplementary Material 1 [file 10020_2024_1038_MOESM1_ESM.docx]

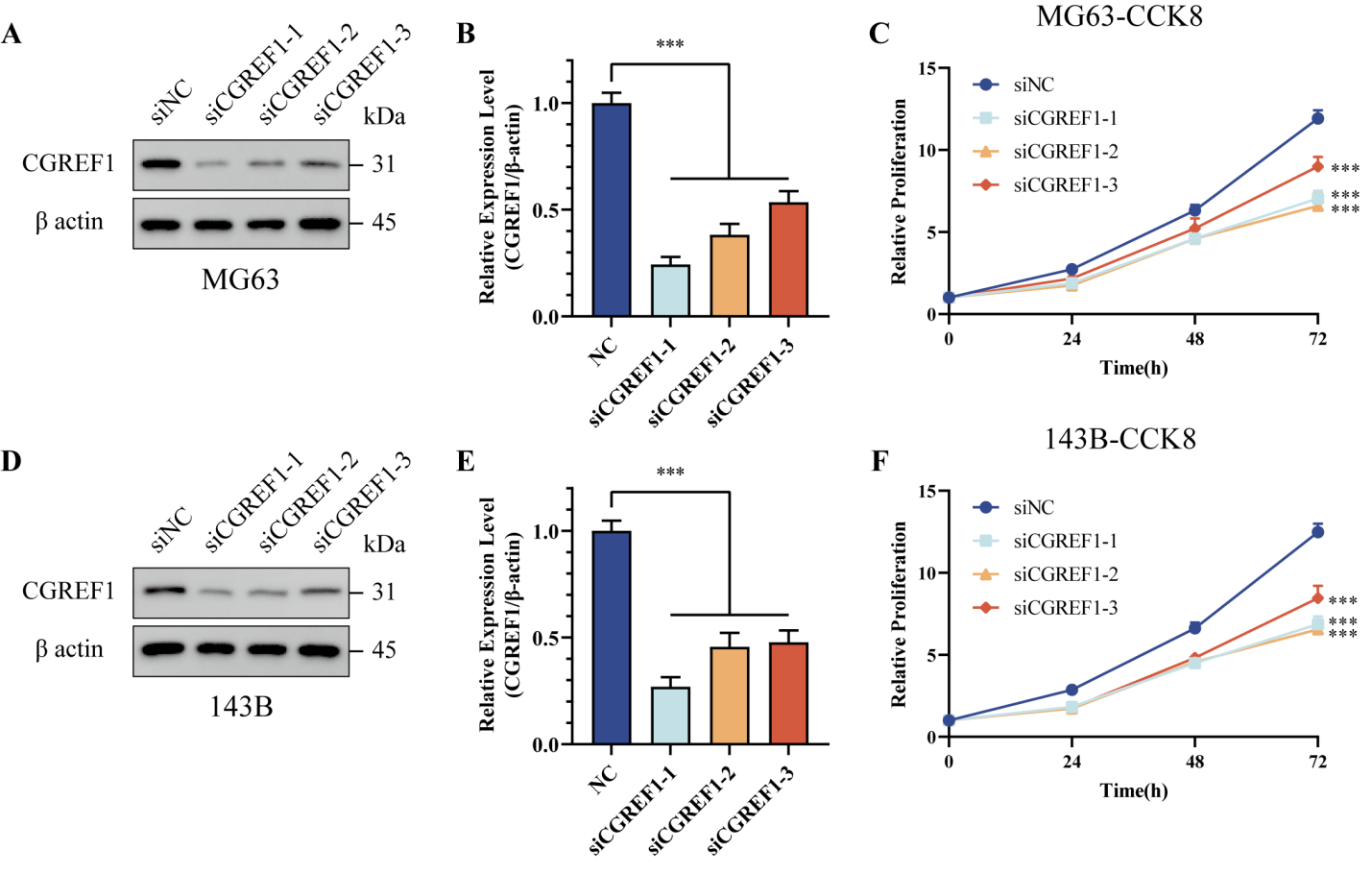
 **Supplementary Figure 1.** (A-B) The Western blot assay was performed to analyze the downregulation of CGREF1 in MG63 cell line after transfection with three siRNAs. The results from three independent experiments were presented concisely in a histogram format. (C) CCK8 assay was used to detect the proliferation ability of MG63 cells after transfection with siRNA. (D-E) The Western blot assay was performed on 143B cell lines after transfection with three siRNA targeting CGREF1, demonstrating a significant decrease in expression levels. (F) CCK8 assay was used to detect the proliferation ability of 143B cells after transfection with siRNA.


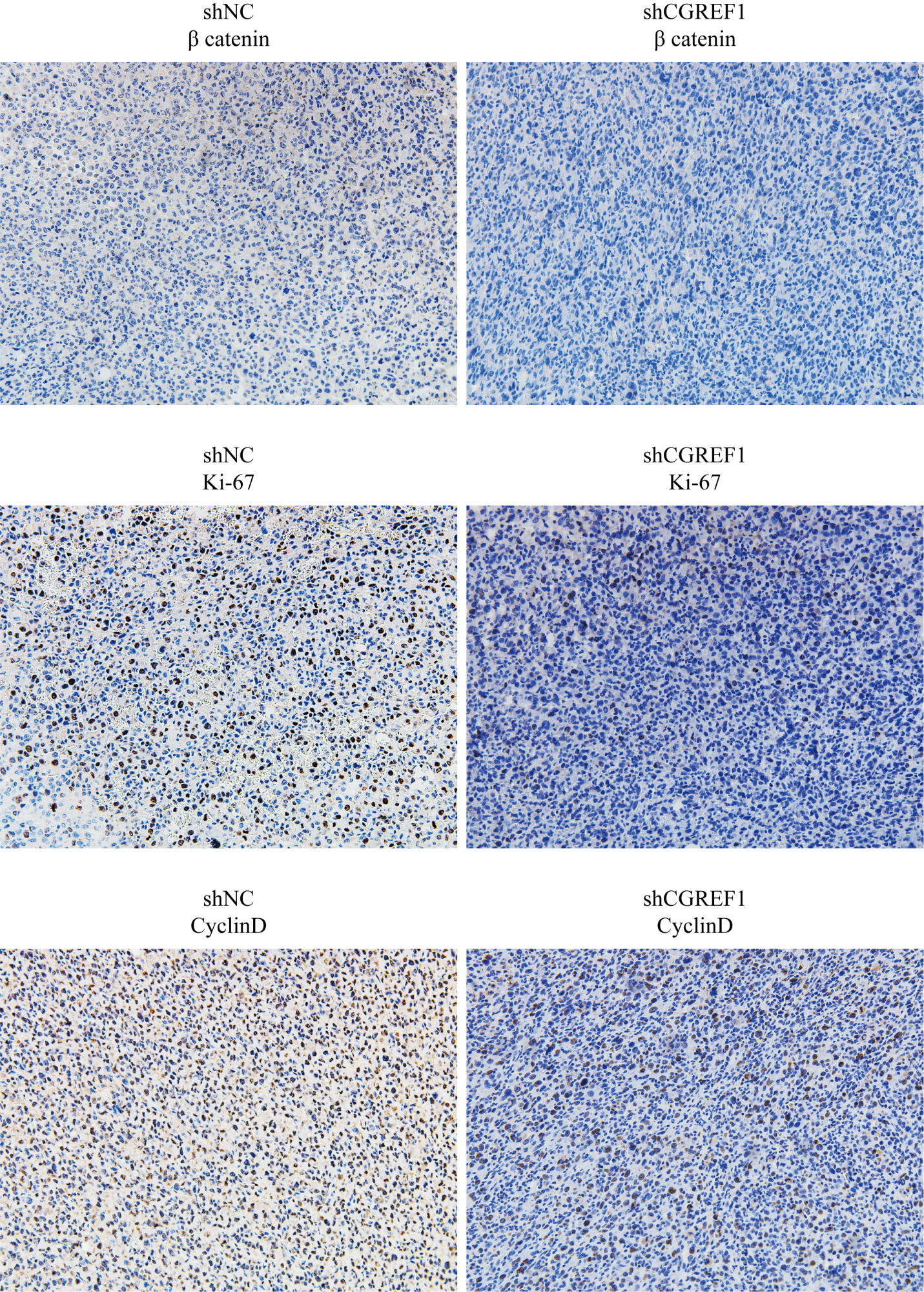


**Supplementary Figure 2.** High-resolution IHC images of β-catenin, Ki-67, and Cyclin D expression in transplanted tumors.
